# Treatment of lichen planus pemphigoides with mesalamine: novel use of this drug^☆^

**DOI:** 10.1016/j.abd.2021.01.011

**Published:** 2022-09-06

**Authors:** Vijay Gandhi, Pradeep Kumar, Ankita Chauhan

**Affiliations:** Department of Dermatology & STD, University College of Medical Sciences and Guru Teg Bahadur Hospital, Delhi, India

Dear Editor,

Lichen Planus (LP) pemphigoides was first described by Kaposi in 1892. It is characterized by widespread bullae formation over the normal skin with preexisting LP.^1^ Autoantibodies IgG formed against BPAg2, BPAg1 and 200kd antigen leads to the development of LP pemphigoides. Treatment includes dapsone, systemic steroids, cyclosporine, azathioprine, and more recently biologics including ustekinumab with variable success rates.^2^, ^3^, ^4^ We report a case of LP Pemphigoides successfully treated by mesalamine as novel use of an older drug.

A 58-year-old male presented with multiple violaceus, pruritic papules, and plaques over dorsal surface of hands and feet for the last 5 years (Fig. 1A), along with oral-genital involvement. Recurrent crops of multiple vesiculobullous lesions were noticed over non-lesional normal skin predominantly over extremities and a few over the trunk (Fig. 2), for 6 months. No vesiculobullous lesions were seen over preexisting hyperpigmented plaques. He was a known patient with Diabetes mellitus on treatment with oral Metformin. Tzanck smear from bullae revealed predominant polymorphonuclear neutrophils. Biopsy from the hypertrophic plaque showed hyperkeratosis, basal cell vacuolation and lymphocytic infiltrate (Fig. 1B) whereas biopsy from bullous lesion revealed subepidermal split with neutrophils and few eosinophils (Fig. 3A). Direct immunofluorescence study showed a linear C3 deposit at the dermo-epidermal junction (Fig. 3B). ELISA for BP180 or immunoblotting were not available, so they could not be performed.

**Figure 1:**
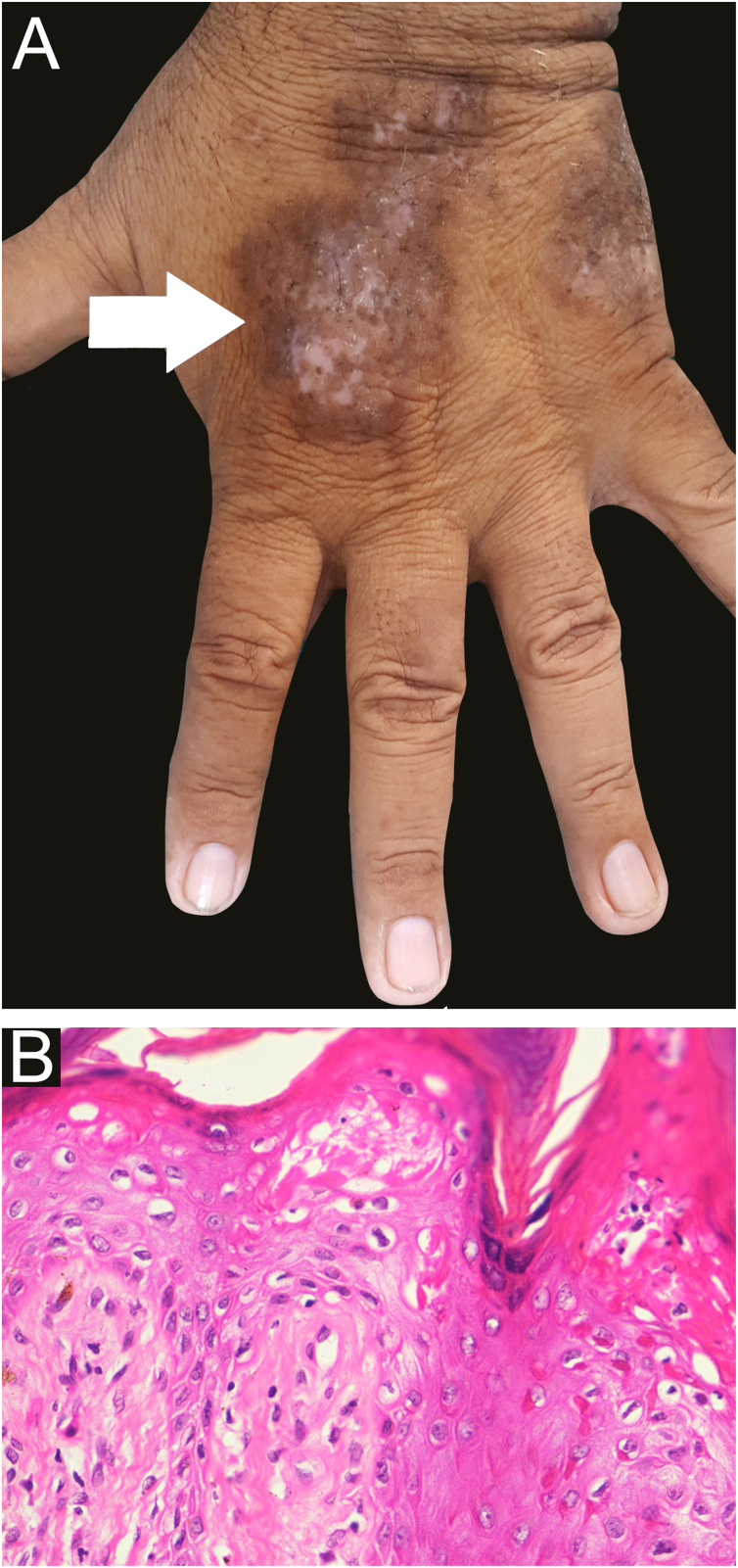
A - clinical image of hypertrophic lichen planus (arrow) B - histopathological examination shows hyperkeratosis, parakeratosis, acanthosis, basal cell vacuolation and lymphocytic infiltrate (Hematoxylin & eosin ×400).

**Figure 2:**
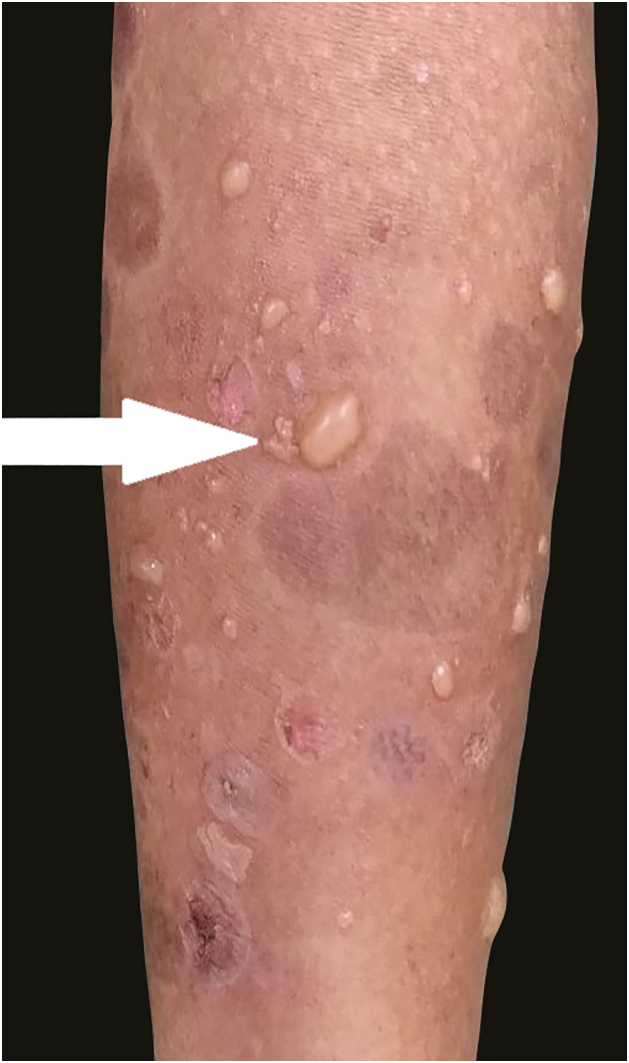
Clinical image of bullous pemphigoid like lesions over normal and inflamed skin (arrow).

**Figure 3:**
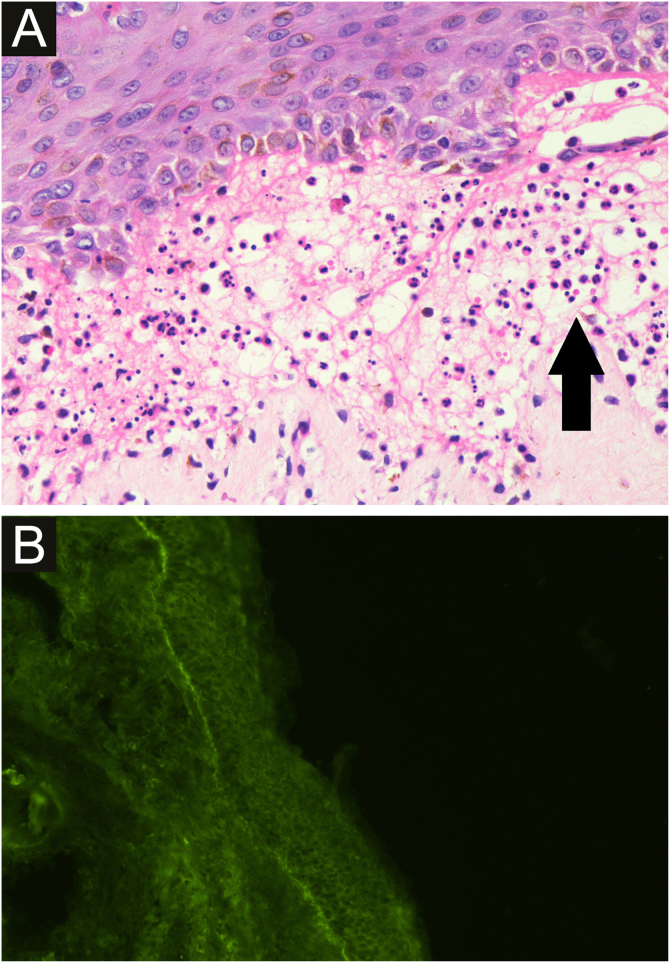
A - light Microscopy from a blister shows subepidermal split with polymorphonuclear neutrophils (black arrow) and few eosinophils (Hematoxylin & eosin stain, ×400), B - direct immunofluorescence shows a linear C3 deposit at the dermo-epidermal Junction while IgA, IgG, IgM, fibrinogen were absent.

A diagnosis of LP pemphigoides was made based on the above findings and he was treated with oral Mesalamine (800 mg thrice daily). The bullous lesions resolved within 2 weeks with no recurrence after 6 months of follow-up. Topical betamethasone dipropionate (0.05%) was used as adjuvant therapy for hypertrophic plaques with gradual regression of lesions.

LP pemphigoides should be differentiated from bullous LP where the bullae arise over preexisting lesions of LP. In contrast to BP which occurs in the elderly, LP pemphigoides are seen to have a younger age of onset, with short-lived bullae, and more neutrophils in subepidermal vesicle on histological examination. DIF study reveals fine, linear, continuous pattern of IgG and C3 deposit at the basement membrane zone (similar to BP) but immune-electron microscopic studies reveal these deposits to be localized to the base of bullae as contrasted to the roof as in BP.

Mesalamine (5 Amino-salicylic acid) is a drug structurally related to salicylates and is used primarily as an anti-inflammatory agent (for ulcerative colitis) and Disease Modifying Anti-Rheumatic Drug for the treatment of Rheumatoid arthritis and other seronegative arthritis. To the best of our knowledge, Mesalamine has not been used for the treatment of LP, bullous pemphigoid, or LP pemphigoides. 2, 3, 4 No major adverse reaction was seen in our patient, although lichenoid eruptions had been reported during sulfasalazine therapy.^5^ Because of Diabetes Mellitus in our patient, we decided to use Mesalamine as it has a favorable effect on the metabolic milieu. We propose Mesalamine as an alternative drug in LP pemphigoides, especially in patients with metabolic syndrome due to its favorable anti-inflammatory effects.

## Financial support

None declared.

## Authors’ contributions

Vijay Gandhi: Concepts; design; definition of intellectual content; literature search; clinical studies; manuscript preparation; manuscript editing; manuscript review; guarantor.

Pradeep Kumar: Concepts; definition of intellectual content; clinical studies; manuscript editing; manuscript review.

Ankita Chauhan: Concepts; definition of intellectual content; clinical studies; manuscript editing; manuscript review.

## Conflicts of interest

None declared.
